# “Sometimes you have to take the person and show them how”: adapting behavioral activation for peer recovery specialist-delivery to improve methadone treatment retention

**DOI:** 10.1186/s13011-023-00524-3

**Published:** 2023-03-06

**Authors:** Mary B. Kleinman, Morgan S. Anvari, Valerie D. Bradley, Julia W. Felton, Annabelle M. Belcher, C. J. Seitz-Brown, Aaron D. Greenblatt, Dwayne Dean, Melanie Bennett, Jessica F. Magidson

**Affiliations:** 1 Department of Psychology, University of Maryland at College Park, College Park, Maryland, USA; 2 Center for Health Policy and Health Services Research, Henry Ford Health System, Detroit, MI USA; 3 Department of Psychiatry, University of Maryland School of Medicine, Baltimore, MD USA

**Keywords:** Peer recovery specialist, Behavioral activation, Methadone, Opioid use disorder, Health disparities

## Abstract

**Background:**

Despite efficacy of medication for opioid use disorder, low-income, ethno-racial minoritized populations often experience poor opioid use disorder treatment outcomes. Peer recovery specialists, individuals with lived experience of substance use and recovery, are well-positioned to engage hard-to-reach patients in treatment for opioid use disorder. Traditionally, peer recovery specialists have focused on bridging to care rather than delivering interventions. This study builds on research in other low-resource contexts that has explored peer delivery of evidence-based interventions, such as behavioral activation, to expand access to care.

**Methods:**

We sought feedback on the feasibility and acceptability of a peer recovery specialist-delivered behavioral activation intervention supporting retention in methadone treatment by increasing positive reinforcement. We recruited patients and staff at a community-based methadone treatment center and peer recovery specialist working across Baltimore City, Maryland, USA. Semi-structured interviews and focus groups inquired about the feasibility and acceptability of behavioral activation, recommendations for adaptation, and acceptability of working with a peer alongside methadone treatment.

**Results:**

Participants (*N* = 32) shared that peer recovery specialist-delivered behavioral activation could be feasible and acceptable with adaptations. They described common challenges associated with unstructured time, for which behavioral activation could be particularly relevant. Participants provided examples of how a peer-delivered intervention could fit well in the context of methadone treatment, emphasizing the importance of flexibility and specific peer qualities.

**Conclusions:**

Improving medication for opioid use disorder outcomes is a national priority that must be met with cost-effective, sustainable strategies to support individuals in treatment. Findings will guide adaptation of a peer recovery specialist-delivered behavioral activation intervention to improve methadone treatment retention for underserved, ethno-racial minoritized individuals living with opioid use disorder.

**Supplementary Information:**

The online version contains supplementary material available at 10.1186/s13011-023-00524-3.

## Background

More than two million people are living with an opioid use disorder (OUD) in the US, with only about 20% receiving treatment in the past year [1]. Deaths associated with the opioid epidemic continue to rise, especially among Black and African American individuals living with OUD [2, 3]. Greater attention and resources are needed to focus on providing ethno-racial minoritized individuals accessible and culturally acceptable treatment and recovery support.

Medication treatment for opioid use disorder (MOUD), including methadone, buprenorphine, and naltrexone, is an effective component of treatment for OUD [4, 5]. While effective for those who stay in treatment, MOUD programs typically show low retention rates, below 50% at six months post treatment initiation [6–8], with ethno-racial minority status predictive of poor retention [9–11]. This may be in part attributable to systemic barriers and structural racism that make it particularly difficult for Black and African American patients to receive and remain adherent to MOUD, such as racism within the healthcare system and strict program policies [12, 13].

Peer recovery specialists (PRSs), persons with lived experience of OUD and recovery, may be particularly well suited to support patients’ retention on MOUD. PRSs typically provide a range of services, such as linkage to resources and health care navigation, and may also have a unique opportunity to decrease stigma-related barriers through shared experience [14, 15]. PRS services have been shown to reduced substance re-use; improve relationships with treatment providers; increase treatment retention and patient satisfaction; and reduce costly acute care utilization [16, 17]. However, few studies have specifically investigated the role of PRSs in supporting MOUD.

While PRSs are typically trained in motivational interviewing and to support linkage to other resources, their training in and delivery of other evidence-based interventions (EBIs) has been limited [18, 19]. However, building off of work in low- and middle-income countries that have relied on task sharing (i.e., peer and lay health worker delivery of EBIs for SUD and mental health [20]), there also are opportunities to also explore PRS-delivered EBIs in the US. This approach could increase accessibility to EBIs in settings serving low-income and ethno-racial minoritized populations, while also potentially supporting the expansion, reimbursement, and funding of PRS services.

Based on prior research, a behavioral intervention that targets increase in positive reinforcement may be the most promising for improving MT retention. Although contingency management interventions (i.e., escalating monetary incentives for continued negative urinalysis or retention) has empirical support [21], there has been low adoption in clinical settings given provider and organizational barriers, including cost and clinician ideology (i.e., clinicians’ views that contingency management may undermine intrinsic motivation and does not address underlying factors contributing to use) [22]. Yet, the existing empirical support for contingency management demonstrates the role of targeting positive reinforcement to improve MOUD retention. Given the barriers to implementing contingency management, it is critical to identify alternative reinforcement-based approaches that can be scaled rapidly and sustained in community-based settings. One intervention found to be particularly suitable for PRS delivery is behavioral activation (BA), which builds on natural reinforcement in people’s environments [23–25]. Although originally developed for depression, BA has gained empirical support in improving SU treatment outcomes by increasing behaviors that bring pleasure and mastery and continued substance-free positive reinforcement [26, 27]. BA has been regarded as highly appropriate in resource-limited settings globally [23, 28, 29], as well as an effective, feasible and acceptable intervention to reduce SU [26] and improve SU treatment retention [27] in the US, including with PRS delivery to support linkage to SU treatment [25].

Though PRS-delivered BA for SU has gained empirical evidence in underserved settings both locally and internationally, there have been limited efforts to explore PRS-delivered BA to support OUD outcomes specifically. Given prior evidence that BA can support retention in SU treatment [27], it may be a particularly relevant approach for improving methadone retention and reducing barriers to retention. The purpose of this study was to gather stakeholder feedback to guide adaptation of a PRS-delivered BA intervention to improve methadone retention and address barriers and facilitators to implementation in methadone treatment. This study intends to build off of an existing BA intervention that was previously developed for peer delivery to support engagement in treatment for SUD [25, 30]. Specifically, the aims of this study were to: 1) assess perceptions of the appropriateness and acceptability of working with a PRS to promote MT retention; 2) assess perceptions of the appropriateness and acceptability of a BA intervention in the context of MT; 3) identify barriers and facilitators to effective implementation of a PRS-delivered BA intervention in the OTP setting; and 4) solicit suggestions for adaptations to BA content and/or structure to further improve appropriateness for this setting and population.

## Methods

We utilized the Standards for Reporting Qualitative Research to structure this manuscript [31].

### Setting

This study took place at a community-based opioid treatment program (OTP) in Baltimore City, Maryland, USA. The program is certified by the Maryland Department of Health and Commission on Accreditation of Rehabilitation Facilities and serves over six hundred patients receiving methadone.

### Participants

Participants for this study (*N* = 32) included program providers, staff, and local PRSs (*n* = 12) and patients (*n* = 20) receiving methadone, purposefully sampled who were successfully engaged in treatment as well as those who were struggling with retention. The majority of participants identified as male (59.4%) and Black or African American (65.6%) with a mean age of 48.7 (*SD* 10.1) across patients, staff, and PRSs. Demographic and other characteristics can be found in Table 1.

**Table 1:** Participant demographics and other characteristics

	Patient Participants (*n* = 20)	Staff/PRS Participants (*n* = 12)	Total Participants (*n* = 32)
	*n* (%)	*n* (%)	*n* (%)
Race			
Black or African American	12 (60.0)	9 (75.0)	21 (65.6)
White	6 (30.0)	2 (16.7)	8 (25.0)
Other	2 (10.0)	1 (8.3)	3 (9.4)
Male gender^a^	14 (70.0)	5 (41.7)	19 (59.4)
Female gender^a^	6 (30.0)	7 (58.3)	13 (40.6)
Mean age (SD)	48.4 (10.0)	49.2 (0.7)	48.7 (10.1)
Highest level of education			
Some high school	7 (35.0)	0 (0.0)	7 (21.9)
High school diploma or GED	8 (40.0)	3 (25.0)	11 (34.4)
Some college	3 (15.0)	1 (8.3)	4 (12.5)
Associate’s degree	2 (10.0)	3 (25.0)	5 (15.6)
Bachelor’s degree	0 (0.0)	2 (16.7)	2 (6.3)
Master’s degree or higher	0 (0.0)	3 (25.0)	3 (9.4)
SUD history	20 (100)	10 (83.3)	30 (93.8)
Mean age of first SU (SD)^b^	17.7 (5.1)	NA	–
Avg years working in SUD treatment (SD)^c^	NA	9.6 (7.6)	–

*SD* Standard deviation, *SU* Substance use, *SUD* Substance use disorder, *PRS* Peer recovery specialist

^a^Participants self-identified as male or female gender though non-binary and other gender responses were included as options on the demographics survey

^b^Question only asked of patient participants

^c^Question only asked of staff and PRS participants

Program staff were purposefully selected to represent a range of roles in patient care and program administration, including one staff member who also works as a PRS. In our purposeful recruitment strategy, we recruited staff members at the OTP who were directly responsible for patient care and worked directly with patients who were at risk for or struggling with poor retention. We also aimed to have multidisciplinary representation across counseling, nursing/medical, and social work. We recruited PRSs who work across a range of recovery support services in Baltimore City through networking with a peer research collaborator. Program staff participants included five addiction counselors, one nurse, one social worker, and one PRS who described their role as “peer coach.” PRS participants worked across a range of roles: peer coach, state-level program administration, and “residential coach,” and “other counseling.”

In addition to recruitment with flyers and word of mouth, patients were referred by MT program staff. To recruit patients needing support with retention, staff identified individuals who had missed at least five MT doses in the past two weeks, based on dosing records. As recommended in qualitative analysis, sample sizes reflect the number of individuals needed to reach theoretical saturation [32]. Preliminary coding was conducted in real-time to assess for theoretical saturation. We continued recruiting participants for both focus groups and individual interviews until no new ideas were emerging related to primary study aims across responses.

### Procedures

Conducting both focus groups and individual interviews allowed us to capture the patient, staff, and PRS perspectives. We opted to include different data collection methods (individual interviews and focus groups) to promote comfort and participation, and potentially reduce stigma, as suggested by collaborators who have been involved in other research projects at this site. Focus groups included a maximum of six participants in each. All participants chose to participate in a focus group or individual interview, but could not participate in both. All participants provided written informed consent before taking part in a focus group or interview. Focus groups were separated such that patients only participated in groups with other patients. Staff and PRSs participated in focus groups together. Twenty-two participants took part in focus groups (11 provider focus group participants and 11 patient focus group participants) and ten participated in interviews (one provider interview and nine patient interviews). We conducted three provider focus groups and two patient focus groups.

All participants received $25 gift card compensation for their time. All study procedures were approved by the University of Maryland, College Park Institutional Review Board with Interagency Agreement approved by the University of Maryland, Baltimore. Interviews and focus groups were conducted between September 2019 and March 2020.

To solicit feedback on implementation of PRS-delivered BA to support MT retention, interviews and focus groups followed semi-structured guides developed both with stakeholder feedback and following the Assessment-Decision-Administration-Production-Topical Experts-Integration-Training–Testing (ADAPT-ITT) model, an empirically supported framework for adapting psychotherapies [33]. Specifically, this qualitative phase focused on Assessment, Decision, and Administration. Stakeholders involved in interview guide development included OTP leadership, collaborators who conduct research at the same OTP, and people who are involved in PRS training in Maryland. Interview and focus group guides included the same questions for each group, respectively. The patient guide asked about their perception of working with a PRS and BA content while the staff/PRS guide asked about how their patients would engage with this type of intervention. Within the staff/PRS guide, certain questions and probes were directed towards staff and PRS participants separately. For example, we asked PRS participants to describe anything in their current work with patients that is similar to the Behavioral Activation approach. Interview guides for patients and staff/PRSs can be found in Appendix A. Participants were asked about the appropriateness and acceptability of working with a PRS to promote MT retention. We based our definitions of appropriateness and acceptability on Proctor’s definitions of implementation outcomes [34]. Appropriateness was defined as perceived fit and relevance of working with a PRS in a methadone treatment setting and usefulness/relevance of core BA content (scheduling substance-free, rewarding activities). Acceptability was defined as satisfaction with the concepts of working with a PRS interventionist and core BA content. Participants were also asked to identify barriers and facilitators to implementing a PRS-delivered intervention in this setting, including suggestions for adapting BA to further improve appropriateness for this setting and population. Guided by ADAPT-ITT, participants were briefly introduced to BA and asked to identify substance-free, rewarding activities and potential facilitators and barriers to those activities. Additionally, participants were asked for feedback on group versus individual intervention sessions, including group BA activities in addition to specific recommendations about location, timing, and duration of BA intervention. Interviews and focus groups were audio recorded.

### Analysis

Recordings were transcribed and reviewed for accuracy. The coding team iteratively developed patient and staff/PRS codebooks outlining themes, sub-themes, and definitions in the transcripts [35]. Using these codebooks, two independent researchers (undergraduate research assistants) coded transcripts using Nvivo v.12. Coders met weekly to discuss and resolve discrepancies as well as modify and add new codes to the codebook. A third-person arbiter (doctoral student research assistant) was involved in meetings and resolution of coding discrepancies and decisions about definitions of new codes were made by discussion and consensus. Transcripts were analyzed with codebook/template thematic analysis [36] to both deductively analyze themes from the interview guide while inductively identifying new ideas. A summary table of primary themes and related codes pertaining to this analysis can be found in Appendix B. Deductive codes provided an overarching framework guided by ADAPT-ITT for intervention adaptation, whereas the specific feedback and ideas for adaptation were inductively coded. For example, inductive codes included peer leading/ doing activities with patients, the need to allow flexibility in the intervention delivery, and patients wanting/needing something to fill their time or deal with boredom.

## Results

Intersecting themes were conceptualized following our four study aims. Participants expressed perceived acceptability of the PRS role and appropriateness for the OTP setting (Aim 1 – acceptability and appropriateness of PRS role). Participants identified activity scheduling as relevant and described aspects of BA that are appropriate and acceptable for the OPT setting and MT patient population (Aim 2 – acceptability and appropriateness of BA). Participants described barriers to implementation, including: balancing priorities, time, and program requirements; challenges associated with activity engagement; and inconsistent level of interest or motivation (Aim 3a – Barriers). Participants also provided input on how to navigate barriers and support acceptability, namely by considering optimal timing of intervention sessions and allowing for flexibility (time, location, frequency) (Aim 3b – Facilitators). Finally, participants provided insight on adapting and implementing BA for this setting through group work and/or PRS-coordinated or guided activities (Aim 4 – BA adaptation).

### Acceptability and appropriateness of the PRS role and value of working with a PRS

Staff and patients described the acceptability and appropriateness of the PRS role in the methadone treatment context. Participants noted that qualities unique to a PRS, such as their shared experience, may help “reach the client in places that a counselor may not” [staff participant]. This relatability was also described as motivating for patients who look at the PRS as a role model. Participants noted that having a peer with lived SU experience would allow them to envision themselves in active recovery, with their peer inspiring motivation:

*“Tell me how you did it, man. Tell me how you sustained your cleanness time. You know what I mean? Hearing it from him, it’s going to mean a lot to me. You know what I mean? And...I can look at him and say okay, if he can do it, I can do it.”* [patient participant]

Participants shared that a peer’s lived SU experience would be invaluable in comparison to someone who learned about SU through formal education:

*“A lot of times it helps if they walked in my shoes. You know, because we have some people that just go to school for it that want to help, because they have a family member, or mom or dad who was an addict. You know, a lot of times it helps more if the person themself experienced it.”* [patient participant]

While most participants regarded shared experience as a highlight of working with a peer, one participant described the potential risk for peer over-identification with patients. They describe how this situation could begin to blur boundaries:

*“The peers identified a little too much to the point that next thing the peer becomes like the client. You know, and doing activities and stuff, I would, I'm just saying, I would want to make sure that the peers because sometimes with the peers, they also forget that they have the help because they take on the problems of our clients.”* [staff participant]

This participant’s comment highlights potential challenges that peers face in maintaining appropriate boundaries with their clients and attending to self-care.

Personal and professional qualities were shared as desirable to promote acceptability, including, but not limited to: authenticity, professional certification, trustworthiness, and shared gender identity as the participant. Patient participants mentioned that they would want a PRS to be themselves around clients and act “like they would act any other time.” A staff interview participant shared that she thinks that peers need to be able to “interact with patients in a way that is very blunt and direct.”

Some staff and PRS participants reported a preference for professional certification among peers working in this type of role:

*“I just believe in credentialled professionals. That is not to take anything away from a person that is not a credentialled peer, it's just training enhances skill delivery.”* [PRS participant]

Meanwhile, patients mentioned wanting a PRS to have relevant education related to recovery but did not mention professional certification.

Some participants (staff, PRS, and patients) expressed that shared gender identity between patients and peers would be important. For example,

*“Some guys will come to talk to females, but a lot of females clients don't feel as comfortable with some personal things they might want to talk to about to a man, you know, they prefer a female.”* [PRS participant]

On the other hand, several patient participants said that gender and other demographic similarities “don’t matter.”

Patient participants noted that they would be comfortable with a PRS working in collaboration with other members of their treatment team. PRS’ familiarity with community resources and social services was described as having potential to take work off the plates of treatment staff and elevate services being delivered to patients. Moreover, patients explained that PRS’ credibility from their shared experience can make patients more willing to listen to and believe a PRS than other treatment staff.

Due to their integration within the community and approachability, PRSs were described as serving a vital role in engaging patients. One staff member explained that in their experience, without the peer on their team, their attendance “would still be zero.” While a majority of responses to integrating a PRS into larger treatment context indicated acceptability, it is important to note that some staff and PRS participants offered feedback on the definition and perceived boundaries of the PRS role. Some expressed concern about the clinical scope of PRS work and capacity for working with patients with mental health conditions. One staff participant stated:

*“I think you really have to be experienced in that profession to really deal with certain person's mental disorders than just being a peer…I just think…that's not their level of skills. And I just don't think I would, me personally, I would put them up to doing that.”*

Maintaining expectations of a PRS interventionist that are in-line with the peer role was described by staff and PRSs as important.

### Barriers to PRS-delivered BA in community-based MT

Participants identified several potential barriers to engaging with a PRS more broadly and PRS-delivered BA specifically. First, participants shared details about a number of competing priorities on patients’ time and energy that, though this concern was not specific to BA, they felt would make it difficult for patients to consistently engage with a PRS. One patient participant explained, “I don't do anything for myself. I'm always working-my, whole day is consumed up until late at night.”

Some of the competing demands also included drug treatment program expectations, such as strict dosing times and required meetings with counselors or other treatment staff. As one staff member described it,

*“Sometimes our clients feel like that we have too many [requirements]…we look at the system and see they have a hold on for this, that, and another. Whether it's to see me, see the doctor, see finance, you know, and then also, for someone who has to do their required groups or the groups with their counselor...Hopefully someone that's motivated to actually do it…but adding another thing really depends on the individual.”*

When patients arrive for dosing at the OTP, they often have a list of things that they need to do either before they receive medication or before they can leave. Both patients and staff acknowledged that this can feel overwhelming.

Provider participants also described potential barriers associated with patient motivation for change, as well as maintaining patient motivation and interest in PRS-delivered intervention engagement:

*“Peer support is not the person come and motivate somebody to go to treatment. We're not pushers. So that's going to be your first criterion for somebody that would be successful. They're self-motivated to be in the program.”* [PRS participant]

Participants shared that, while motivation may fluctuate for individuals over time, they felt that the described BA program would be best suited for patients who are motivated and ready for some kind of change. A PRS participant indicated that the BA framework specifically could help support motivation because “it gets their attention and you are able to…take them out of what they used to do” through activity scheduling and engagement.

#### Subtheme- barriers regarding BA specifically

When asked what may get in the way of engaging in substance-free, rewarding activities as part of BA, some participants noted a lack of accessibility due to cost, physical health issues and transportation. Regarding cost, one patient participant said “it’s really a lack of funding of why a lot of people don’t do a lot of things.” Regarding physical health, a PRS participant shared his experience,

*“Some of the clients that I've come across have severe physical disabilities from using [substances]. And some of those things that you talked about, they would need special accommodations for those type of clients.”*

Participants also noted that SU itself would get in the way of engaging in rewarding activities: “Drugs, using will pretty much get in the way and put that all on hold. You come to a complete dead stop” [patient participant]. It was also noted that negative social influences keep some from engaging in positive activities. Relatedly, participants identified experiencing cravings as a barrier to activity engagement, such that cravings bring SU to the forefront of their attention and overshadow other interests:

*“All other things that you like to do get pushed on the back burner, so to speak.”* [patient participant]

Lastly, participants identified not feeling safe or welcome in places where they would do activities, citing concern about crime and discomfort with police presence in some locations.

### Facilitators of PRS-delivered BA in community-based MT

In addition to barriers identified above, participants shared some important factors that would serve as facilitators of a PRS-delivered intervention for the population receiving MT. None of the facilitators identified from participant responses were specific to PRS-delivered BA, but rather any structured intervention delivered by a PRS in this setting.

Participants emphasized the importance of timing when connecting someone to the PRS-delivered intervention. For instance, one participant shared: “If you try to motivate them to do it sooner [than when ready], they gonna feel as though it’s too pushy” [patient participant]. Some participants shared that early in treatment could be helpful for a PRS to work with patients because that is a time when additional support is particularly needed.

*“But in that 30 days time, while they trying to get them on a suitable dosage [of methadone], there's probably going to be usage [of substances] somewhere in there. So, if the guy got somebody to turn to, that's on his feet. You know what I mean? That's supporting them and showing them how to do other things…It will be effective.”* [patient participant]

However, others shared that MT dose stability was important before introducing the BA intervention. So, starting regular intervention sessions with a PRS might not be desirable “until they get their dose right” (patient participant).

When asked about intervention delivery, participants expressed a need for flexibility and tailoring to the individual. One patient participant said, regarding time spent talking with a PRS in an intervention session: “I may only want to talk for half an hour, you know what I'm saying? And then I might have days when I want to talk forever.” Thus, flexibility in duration of intervention sessions may be important. A staff participant emphasized the overall treatment center’s focus on the importance of meeting patients ‘where they are’ in terms of these preferences. Furthermore, a PRS participant responded that this fits with the peer role in that “flexibility is the key as far as being a peer.” Although these facilitators were not specific to BA, these are important suggestions in considering adapting BA (and other more structured interventions) for PRS delivery.

Participants generally indicated that the treatment center was appropriate for holding sessions. Some suggested other locations like meeting outside when weather permits or going “somewhere of interest- a museum, anything where you could walk and talk” [patient participant]. Although, when talking about sensitive, personal topics, general consensus was that private space is important.

Multiple participants recommended small incentives to support engagement:

*“It seems small, but just little things encourage to you to do well, even if they start out just doing it for that reason, eventually, it'll become repetitious and they'll start doing it for themselves.”* [patient participant]

Some incentive ideas shared by participants included food, coffee, raffles/prizes, gift cards, and personal items like “blush, nail polish, a bar of soap” [staff participant].

### Acceptability and appropriateness of BA

Participants described the appropriateness and acceptability of the core of BA—scheduling meaningful, substance-free activities. One participant shared that re-connecting with what made them happy prior to using would help facilitate engagement in substance-free activities:

*“Just touching back into what they their likes are, what their hobbies are, and tap into that and what they used to like to do before they got high and give them a touch of that and it'll probably trigger something, it usually does. And then they'll say, "dang I used to…when I was clean, I used to like to do such and such."… But it's always still there. I think people need to tap into that other side to help them remember.”* [patient participant]

Participants in a staff and PRS focus group noted that engaging in positive activities would also allow people to feel a sense of accomplishment that could translate to other areas of their life. Another participant explained that abstinence from drugs and activity engagement would help them get back in touch with their life: “Escaping back to being me. You know, that familiar place that was always there” [patient participant].

Participants described that engaging in BA would help them fill their time, and ultimately help them achieve better treatment outcomes:

*“I just need something to do with this time of mine, you know what I mean. I get so frustrated with that…I just need something to do to absorb my time.”* [patient participant]

Boredom was identified as a large barrier to successful treatment outcomes; multiple participants said that keeping occupied and not having idle time was a facilitator of successful treatment outcomes.

One staff participant noted that engaging in activities with patients is something that their patients already ask of this staff member, but this is outside of their role in the treatment center. Another participant said this was something they already viewed as part of the PRS role, suggesting feasibility and acceptability of the approach:

*“It's already happening. What you're describing is the central core function of what a peer does. They work with individuals to identify recovery barriers, identify resources to get through those recovery barriers, and then to support that person as they work through that.”* [PRS participant]

Altogether, feedback indicated appropriateness and acceptability of BA intervention content both for patient engagement and PRS delivery.

### Proposed adaptations to BA

Regarding group vs. individual sessions, some patient participants noted the value in sharing one’s own and hearing others’ stories, which would let participants “see that you all relate on one common cause and that's to stay clean” [patient participant]. Staff likewise supported group intervention sessions, stating that they believed fostering a group bond among patients and understanding that they are not alone would facilitate positive treatment outcomes. However, other staff and patients expressed concerns about confidentiality and decreased comfort. Multiple participants expressed interest in a combination of individual and group intervention sessions. Although feedback was mixed on the use of group intervention sessions, a majority of participants regarded the availability of group activities as a potential facilitator of engaging in BA. Patient participants expressed interest in being able to find others with similar interests and doing activities together as a group.

Participants also shared ways that it would be helpful for a PRS to facilitate activities, including: learning from the PRS, having the PRS lead or do the activity with patients, and/or facilitate activity planning. Leading or doing activities with participants was regarded as an opportunity for the PRS to incorporate their lived experience into the intervention, and share activity ideas with participants in which the PRS engages. One patient participant highlighted the importance of this by explaining that many activities would be novel in this community:

*“Well, you know, sometimes you got to take the person and show them how to get there, show them different things. There's a lot of people that haven't even experienced things like that.”*

Patients and staff also indicated that it would be helpful to have someone in charge of establishing a plan for the activities. One staff participant said:

*“Anytime that you're going to identify an opportunity for somebody to take advantage of, is making sure that those resources like transportation and that, are pre-identified. So, making sure you identify what bus routes are available to get to those places, identifying when organizations have opportunities for financial cost reduction.”* [staff participant]

Another staff participant identified program coordination by the PRS as a facilitator of group activities and cohesion, such that program coordination should include connecting patients with similar interests to allow for group activity engagement.

## Discussion

The overall aim of this study was to understand the appropriateness and acceptability of a PRS role to promote methadone treatment retention in a community-based OTP, identify the barriers and facilitators to implement a PRS-delivered BA intervention, and solicit feedback on how we may adapt BA to further improve appropriateness for PRS delivery in this setting. Findings highlight perceived acceptability and appropriateness of PRS work to support patients in MT. Overall, participants described their belief in the utility of a PRS-delivered BA intervention to promote meaningful, substance-free activities. Participants also described potential barriers to consider both in the general implementation of a PRS-delivered intervention and BA-specific content. Important facilitators that could offset those barriers were identified by participants, as well as recommended adaptations to the BA intervention for this setting and population.

PRS models have scaled nationwide in a variety of settings to increase motivation for recovery, support service navigation, promote retention in care, and reduce barriers to engagement [16, 19]. This is due to the limited number of specialized, highly trained mental health providers in community-based settings [37], disparities in access to care for ethno-racial minoritized individuals [38], and SU stigma and low motivation as barriers to engaging in treatment. Despite the recent increase in use of PRSs and their promise for supporting retention, there have been few studies evaluating how PRS-led EBIs may improve MT retention specifically, especially how to adapt an existing EBI for PRS delivery in the OTP context.

Regarding feedback on the PRS role in OTPs more broadly, not specific to BA delivery, participants in our study highlighted many attributes of the PRS role that have been reported as beneficial, such as lived experience with SU and serving as a role model [39]. Although participants did not identify having a shared recovery pathway (e.g., prior experience with MT) as a necessary quality for a PRS working in the context of MT, other feedback suggested that not having shared recovery pathway experience could risk stigmatization towards MT and/or a participant’s chosen recovery path. Nonetheless, it was emphasized by staff participants who were familiar with PRS work and that PRSs are trained to support multiple pathways of recovery. These results contribute important insight into the perceived role of a PRS within the dynamics of an MT care team. Staff participants highlighted the utility of a PRS to better engage patients and serve as a connection to others on the treatment team. They identified this unique role and capability of integrating a PRS into their treatment team as a means of distributing workload and allocating resources more effectively.

One important barrier identified was the boundaries of the peer role. Some PRS and staff participants described a belief that the PRS role and responsibilities should be clearly specified and should be within the peer “lane”, and PRS work should not include clinical assessment or intervention for mental health disorders. This feedback aligns with the PRS role and responsibilities outlined in Maryland’s (and other jurisdictions’) PRS training/certification materials [40]. Given that BA can be perceived as a mental health-oriented intervention, considering these parameters of the PRS role and how interventions such as BA or motivational interviewing fit into that role is essential. However, in low-resource settings globally, there is a precedent for training peers and community health workers in EBIs, including BA [23, 24, 28, 29, 41]. Though we know from other work that it is feasible to train and implement peer-delivered BA interventions, considering the unique licensure and role boundaries in the US will be important for sustainability. Yet, it may also be an opportunity to increase access to evidence-based mental health services for a historically marginalized group with limited access to these services. Finally, feedback on PRS role and concern about taking on complex patient mental health needs highlights the importance of strong supervision and thoughtful support of PRSs, including intentional promotion of self-care and ability to reach out for regular consultation with a trained supervisor.

Patients’ competing responsibilities, such as work and family obligations, were identified as barriers to engaging in a BA intervention. These results are consistent with other research led by our team in a community resource center setting focused on peer linkage to SU treatment, highlighting how competing demands present challenges to engaging in behavioral interventions [25]. Further, in an OTP setting, unique responsibilities related to engaging in MT, often on a daily basis, further compounds this challenge. As patients explained the time demands related to MT requirements and policies, it is important for a PRS to work to identify barriers to MT engagement and collaboratively work with their clients to build strategies to overcome these barriers, for instance through incorporating problem solving techniques into BA. It is important that a PRS-delivered EBI has built-in flexibility, as to not be perceived as an additional restriction on patients’ time that keeps them from reaching their goals (e.g., obtaining employment). It is also important to present the option of intervention engagement at multiple timepoints, accounting for varying and fluctuating readiness to participate based on where an individual patient is in their recovery process. As described by patients, staff, and PRS participants, there is likely not a standard amount of time after MT initiation when patients would be optimally open to the proposed intervention. Previous qualitative findings have similarly pointed to the need for low-barrier participation and openness for re-engagement when implementing a PRS-delivered intervention in a community-based setting [30].

Also consistent with previous research [25], participants identified SU, cravings and feeling unsafe in their environment as barriers to engaging in BA scheduled activities. However, participants also described a preference for a PRS to help schedule, facilitate and even participate in activities with their clients. Active PRS engagement in activity scheduling may be a key component in successfully deploying a BA intervention within this context where various barriers to substance-free, rewarding activity engagement exist. Participants highlighted aspects of BA that they feel are particularly relevant to the patient experience in OUD recovery, focusing on combatting boredom and filling their time. This finding is also in-line with findings from previous research on adaptation of BA for community health worker delivery, targeting SU and HIV medication adherence in South Africa [23, 24, 41]. An important potential benefit of PRS-delivered BA is cost and sustainability, particularly in comparison to contingency management approaches. When considering potential facilitation of activity engagement, it will be important to include costs associated with offering on-site or subsidized activities as well as whether these are sustainably accessible in patients’ lives.

Importantly, patient and provider participants described a need for intervention flexibility in order to be successful in this target population. This will require a careful balance of built-in flexibility to adapt a BA intervention to suit client needs, while also remaining adherent to the core intervention content. Moreover, these reactions highlight that a traditional, 12-week long, weekly BA session structure may not work for all clients, whereas this type of structure may serve as a motivator for others. When directly asked about the proposed length of the intervention (12 weeks) and frequency of meetings with a PRS (weekly), participants all responded favorably without any specific recommendations. Lack of elaboration may reflect participants not having a frame of reference to answer these questions, particularly if they have not previously engaged with a mental health services, PRSs, or more structured interventions. However, in the context of OUD care, participants described that an intervention must remain flexible in order to meet patients where they are, including length. As disengagement with MT is associated with higher rates of morbidity and mortality [42], novel interventions addressing barriers to retention are of high public health importance in confronting the opioid crisis.

### Limitations

Results should be considered in the context of methodological limitations. Though we employed procedures to reach a diverse sample of participants, inclusion criteria required participants to be enrolled in MT. Participant responses were also limited by self-selected, narrow staff representation with only one participant representing medical staff in the MT program. We recognize that a sample size of 32 is not likely to achieve theme saturation within groups (patient/staff/PRS), but the aim of this project was to integrate patient, staff, and PRS responses to the proposed intervention. As a design consideration, it is also important to note that focus groups combined staff and PRS participants. While PRS responses about a peer-delivered intervention could be influenced by staff in other roles within the OTP, we chose to combine provider participants to reinforce the role of a PRS as a member of the healthcare team, a cited challenge in PRS work [14]. Since interviews and focus groups were conducted in the treatment program setting, some participants may have felt uncomfortable sharing their thoughts openly, though researchers did not have any pre-existing relationships with participants. However, all participants spoke and provided feedback in both the focus group and interview contexts. We did not ask about participant reasons for choosing interview versus focus group participation and recommend that for future work that takes a similar approach. We also recognize the limitations to generalizability given this study recruited patients and staff from just one treatment program and one MOUD modality (MT versus other options such as buprenorphine or naltrexone). Finally, while this study focused on a PRS-delivered intervention to support MT retention, other patient-centered outcomes, beyond retention or medication adherence, should also be considered in evaluation of such interventions [43].

## Conclusions

Results suggest potential appropriateness and acceptability of a PRS-delivered BA intervention in MT. Findings are being used to adapt a PRS-delivered BA intervention being piloted to improve MT retention for underserved, ethno-racially diverse (largely Black/African American) individuals with OUD (National Library of Medicine, NCT04248933). This study’s feedback is essential to guide further adaptation and promote feasibility and acceptability of PRS-delivered EBIs, specifically BA. An important component of this adaptation will be considering the boundaries of the PRS role in promoting the sustainability of delivering BA. Findings support the need to conduct the larger trial to evaluate the effectiveness and implementation of this model.

## Supplementary Information

**Additional file 1.** Focus group and interview guides.

**Additional file 2.** Thematic framework.

## Data Availability

The qualitative dataset analyzed during the current study is available from the corresponding author on request.

## Abbreviations

BA: Behavioral activation
EBI: Evidence-based intervention
MOUD: Medication for opioid use disorder
MT: Methadone treatment
OTP: Opioid treatment program
OUD: Opioid use disorder
PRS: Peer recovery specialist
SU: Substance use
SUD: Substance use disorder

## References

[1] Substance Abuse and Mental Health Services Administration. Key substance use and mental health indicators in the United States: Results from the 2021 National Survey on Drug Use and Health (HHS Publication No. PEP22-07-01-005, NSDUH Series H-57). Center for Behavioral Health Statistics and Quality, Substance Abuse and Mental Health Services Administration; 2022. Available from: https://www.samhsa.gov/data/report/2021-nsduh-annual-national-report.

[2] Khatri UG, Pizzicato LN, Viner K, Bobyock E, Sun M, Meisel ZF, et al. Racial/ethnic disparities in unintentional fatal and nonfatal emergency medical services–attended opioid overdoses during the COVID-19 pandemic in Philadelphia. JAMA Netw Open. 2021;4(1):e2034878. 10.1001/jamanetworkopen.2020.34878 PMC7821023 33475751

[3] Patel I, Walter LA, Li L (2021). Opioid overdose crises during the COVID-19 pandemic: implication of health disparities. Harm Reduct J.

[4] Mattick RP, Breen C, Kimber J, Davoli M (2009). Methadone maintenance therapy versus no opioid replacement therapy for opioid dependence. Cochrane Database Syst Rev.

[5] Mattick RP, Breen C, Kimber J, Davoli M. Buprenorphine maintenance versus placebo or methadone maintenance for opioid dependence. Cochrane Database Syst Rev. 2014;2:CD002207. 10.1002/14651858.cd002207.pub4. 10.1002/14651858.CD002207.pub4 PMC10617756 24500948

[6] Timko C, Schultz NR, Cucciare MA, Vittorio L, Garrison-Diehn C (2016). Retention in medication-assisted treatment for opiate dependence: A systematic review. J Addict Dis.

[7] Williams AR, Nunes EV, Bisaga A, Levin FR, Olfson M (2019). Development of a Cascade of Care for responding to the opioid epidemic. Am J Drug Alcohol Abuse.

[8] Stone AC, Carroll JJ, Rich JD, Green TC (2020). One year of methadone maintenance treatment in a fentanyl endemic area: Safety, repeated exposure, retention, and remission. J Subst Abuse Treat.

[9] Manhapra A, Petrakis I, Rosenheck R (2017). Three-year retention in buprenorphine treatment for opioid use disorder nationally in the Veterans Health Administration. Am J Addict.

[10] Proctor SL, Copeland AL, Kopak AM, Hoffmann NG, Herschman PL, Polukhina N (2015). Predictors of patient retention in methadone maintenance treatment. Psychol Addict Behav J Soc Psychol Addict Behav.

[11] Samples H, Williams AR, Olfson M, Crystal S (2018). Risk factors for discontinuation of buprenorphine treatment for opioid use disorders in a multi-state sample of Medicaid enrollees. J Subst Abuse Treat.

[12] Volkow ND, Frieden TR, Hyde PS, Cha SS (2014). Medication-assisted therapies–tackling the opioid-overdose epidemic. N Engl J Med.

[13] Goedel WC, Shapiro A, Cerdá M, Tsai JW, Hadland SE, Marshall BDL (2020). Association of racial/ethnic segregation with treatment capacity for opioid use disorder in counties in the United States. JAMA Netw Open.

[14] Jack HE, Oller D, Kelly J, Magidson JF, Wakeman SE (2018). Addressing substance use disorder in primary care: The role, integration, and impact of recovery coaches. Subst Abuse.

[15] Anvari MS, Kleinman MB, Massey EC, Bradley VD, Felton JW, Belcher AM (2022). “In their mind, they always felt less than”: The role of peers in shifting stigma as a barrier to opioid use disorder treatment retention. J Subst Abuse Treat.

[16] Eddie D, Hoffman L, Vilsaint C, Abry A, Bergman B, Hoeppner B (2019). Lived experience in new models of care for substance use disorder: A systematic review of peer recovery support services and recovery coaching. Front Psycho.

[17] Magidson JF, Regan S, Powell E, Jack HE, Herman GE, Zaro C (2021). Peer recovery coaches in general medical settings: Changes in utilization, treatment engagement, and opioid use. J Subst Abuse Treat.

[18] Bassuk EL, Hanson J, Greene RN, Richard M, Laudet A (2016). Peer-delivered recovery support services for addictions in the United States: A systematic review. J Subst Abuse Treat.

[19] Reif S, Braude L, Lyman DR, Dougherty RH, Daniels AS, Ghose SS (2014). Peer recovery support for individuals with substance use disorders: assessing the evidence. Psychiatr Serv Wash DC.

[20] Magidson JF, Jack HE, Regenauer KS, Myers B (2019). Applying lessons from task sharing in global mental health to the opioid crisis. J Consult Clin Psychol.

[21] Carroll KM, Weiss RD (2017). The role of behavioral interventions in buprenorphine maintenance treatment: A review. Am J Psychiatry.

[22] Carroll KM (2014). Lost in translation? Moving contingency management and cognitive behavioral therapy into clinical practice. Ann N Y Acad Sci.

[23] Magidson JF, Andersen LS, Satinsky EN, Myers B, Kagee A, Anvari M (2020). “Too much boredom isn’t a good thing”: Adapting behavioral activation for substance use in a resource-limited South African HIV care setting. Psychotherapy.

[24] Magidson JF, Joska JA, Belus JM, Andersen LS, Regenauer KS, Rose AL (2021). Project Khanya: results from a pilot randomized type 1 hybrid effectiveness-implementation trial of a peer-delivered behavioural intervention for ART adherence and substance use in HIV care in South Africa. J Int AIDS Soc.

[25] Satinsky EN, Doran K, Felton JW, Kleinman M, Dean D, Magidson JF (2020). Adapting a peer recovery coach-delivered behavioral activation intervention for problematic substance use in a medically underserved community in Baltimore City. PLoS ONE.

[26] Daughters SB, Magidson JF, Anand D, Seitz-Brown CJ, Chen Y, Baker S (2018). The effect of a behavioral activation treatment for substance use on post-treatment abstinence: a randomized controlled trial. Addict Abingdon Engl.

[27] Magidson JF, Gorka SM, MacPherson L, Hopko DR, Blanco C, Lejuez CW (2011). Examining the effect of the Life Enhancement Treatment for Substance Use (LETS ACT) on residential substance abuse treatment retention. Addict Behav.

[28] Patel V, Weobong B, Nadkarni A, Weiss HA, Anand A, Naik S (2014). The effectiveness and cost-effectiveness of lay counsellor-delivered psychological treatments for harmful and dependent drinking and moderate to severe depression in primary care in India: PREMIUM study protocol for randomized controlled trials. Trials.

[29] Patel V, Weobong B, Weiss HA, Anand A, Bhat B, Katti B (2017). The Healthy Activity Program (HAP), a lay counsellor-delivered brief psychological treatment for severe depression, in primary care in India: a randomised controlled trial. Lancet Lond Engl.

[30] Kleinman M, Magidson J, Doran K, Seitz-Brown C, Satinsky EN, Loeb F, et al. Implementing a peer recovery coach-delivered behavioral intervention to support engagement in substance use treatment from a community setting in Baltimore City. In: Proceedings of the Addiction Health Services Research (AHSR) 2020: Virtual Conference. Addict Sci Clin Pract. 2020;15(Supplement 2):A33. 10.1186/s13722-020-00208-4.

[31] O’Brien BC, Harris IB, Beckman TJ, Reed DA, Cook DA (2014). Standards for reporting qualitative research: a synthesis of recommendations. Acad Med.

[32] Pope C, Mays N (2006). Qualitative Research in Health Care.

[33] Wingood GM, DiClemente RJ (1999). The ADAPT-ITT model: a novel method of adapting evidence-based HIV Interventions. J Acquir Immune Defic Syndr.

[34] Proctor E, Silmere H, Raghavan R, Hovmand P, Aarons G, Bunger A (2011). Outcomes for implementation research: Conceptual distinctions, measurement challenges, and research agenda. Adm Policy Ment Health.

[35] Boyatzis RE (1998). Transforming Qualitative Information: Thematic Analysis and Code Development.

[36] Brooks J, McCluskey S, Turley E, King N (2015). The Utility of Template Analysis in Qualitative Psychology Research. Qual Res Psychol.

[37] Health Resources and Services Administration/National Center for Health Workforce Analysis; Substance Abuse and Mental Health Services Administration/Office of Policy, Planning, and Innovation. National Projections of Supply and Demand for Behavioral Health Practitioners: 2013-2025. Rockville; 2015. Available from: https://bhw.hrsa.gov/sites/default/files/bureau-health-workforce/data-research/behavioral-health-2013-2025.pdf.

[38] Lagisetty PA, Ross R, Bohnert A, Clay M, Maust DT (2019). Buprenorphine treatment divide by race/ethnicity and payment. JAMA Psychiatry.

[39] Barker SL, Maguire N (2017). Experts by Experience: Peer Support and its Use with the Homeless. Community Ment Health J.

[40] Maryland Addiction and Behavioral Health Professional Certification Board. Certified Peer Recovery Specialist (CPRS). https://www.mabpcb.com/certified-peer-recovery-specialist-cprs.

[41] Belus JM, Rose AL, Andersen LS, Ciya N, Joska JA, Myers B (2022). Adapting a behavioral intervention for alcohol use and HIV medication adherence for lay counselor delivery in Cape Town, South Africa: a case series. Cogn Behav Pract.

[42] Sordo L, Barrio G, Bravo MJ, Indave BI, Degenhardt L, Wiessing L (2017). Mortality risk during and after opioid substitution treatment: systematic review and meta-analysis of cohort studies. BMJ.

[43] Bradley V, Kleinman M, Greenblatt A, Belcher A, Seitz-Brown CJ, Tralka H, et al. Defining patient-centered successful methadone treatment outcomes among low-income, minority individuals at a community-based outpatient treatment center. Addict Sci Clin Pr. 2020;15(Suppl 2):A26.

