# Supplementary material for: “Sometimes you have to take the person and show them how”: adapting behavioral activation for peer recovery specialist-delivery to improve methadone treatment retention
Source: Subst Abuse Treat Prev Policy. 2023 Mar 6;18:15. doi: 10.1186/s13011-023-00524-3 (PMC9990281; doi:10.1186/s13011-023-00524-3)
Supplement: Supplementary file 1 — Additional file 1. Focus group and interview guides. [file 13011_2023_524_MOESM1_ESM.pdf]

Appendix A
Focus Group and Interview Guides

Staff/PRS Focus Group and Interview Guide

Note: This document is meant to be a guide for the facilitator to structure the focus groups and interviews; however, the facilitator is encouraged to follow the participants responses and probe to gather as accurate a sense of their perspectives as possible. The primary topic areas will stay the same, but the specific questions we expect to change based on information shared during the respective focus groups and interviews.

Feedback on a Behavioral Activation Intervention (Peer Activate)

We want to get your feedback on one idea we have for helping to keep clients engaged in treatment here at the treatment center. We think this is something that could be led by peers to support clients to get to successful treatment outcomes by using something called Behavioral Activation. The idea is that clients are more likely to succeed when they're doing activities that are rewarding and enjoyable to them and do not involve alcohol or drugs. We're hoping to look at how well this works to have peers support clients to do these activities to lead to successful treatment outcomes here. We want to hear your thoughts on this approach.

Hand out BA/ behavioral cycle visual.

BEHAVIORAL ACTIVATION FOR SUBSTANCE USE

BREAKING THE CYCLE OF SUBSTANCE USE

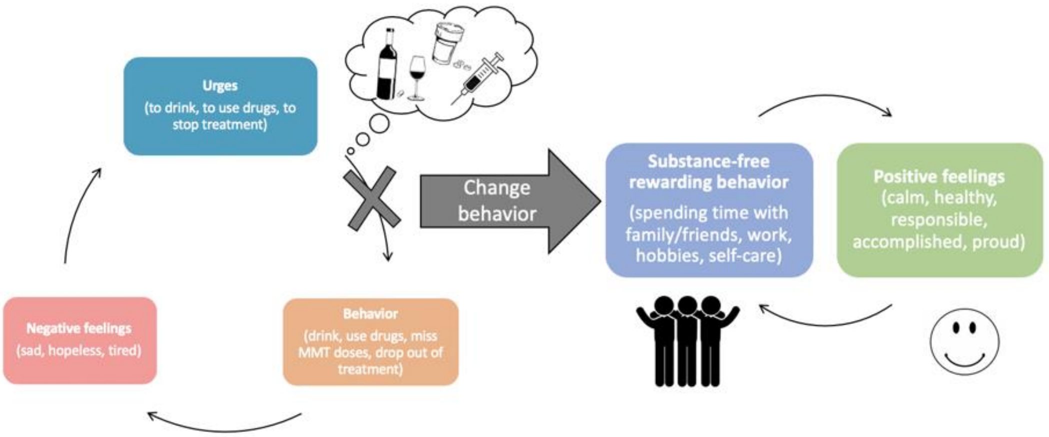

KEY COMPONENTS OF BEHAVIORAL ACTIVATION

- Identifying an individual's **values** in various areas of his/her life (e.g., relationships, health, career)
- Scheduling rewarding, **substance-free activities** that align with these values

We have handed out a simple visual of how Behavioral Activation works. It shows how negative feelings, urges, and behaviors are connected and how we can break the cycle by incorporating positive activities that promote positive feelings.

Do you have any questions about Behavioral Activation right now?

Adaptations to BA for setting

Activity ideas

- In your experience, what things do clients enjoy doing that don't involve substance use?
- What are examples of other activities that people are able to do in the community that you think clients may enjoy doing that do not involve alcohol or drugs?

Barriers and facilitators to activity engagement

- What makes it difficult for clients to take part in these activities?
- What makes it easier for clients to take part in these activities?

  Probe:

  - *How can we make different substance-free activities easier and more accessible?*

Views on peer-led BA at MT initiation

- How can a peer support clients to engage in substance free activities?
- What do you see as the role of a peer in supporting clients to engage in enjoyable, substance-free activities?
- What would it look like to have a peer incorporate their own personal experiences when delivering this approach?

  Probe:

  -How might it work to have peers take part in activities with clients?

What are your thoughts on this type of intervention (that is – peers supporting retention in treatment by engaging in healthy, substance free activities) for clients starting methadone treatment at 1001?

  Probes:

  - *Who do you think this type of intervention would work best for?*
  - *What level of motivation (readiness to recover) would participants need to effectively engage and benefit from this type of intervention?*
  - For PRSs: *Does this intervention sound like something you would be interested in learning to deliver with clients initiating MT? Why or why not?*

Feedback on structure and format

- Would an individual or group peer-led approach to promote substance-free rewarding activities would work better in this setting? Why?

  Probes:

  - *How might group BA therapy (meetings with peers) work in this setting?*

- How might group activities work in this setting?

- Typically this type of approach includes about 10 or 12 meetings. How would that work here?
- How frequently do you think the group should meet? (i.e., once per week? More?) Why?

Probes:

-What would make a weekly session challenging in this setting?

-What would make it easier?

- When would be an ideal time for new patients to start working with a peer to identify rewarding activities? Why?

Probes:

-at MT initiation?

-after maintenance dose is reached?

-how long after MT intake?

- How should scheduling peer sessions fit into a client's dosing schedule and other scheduled time in the methadone program (medical appointments, appointments with counselor, required group meetings)?

Probe:

- For staff: *What does a client's typical day look like just after starting MT?*

Where should meetings with clients take place? What settings make clients most at ease?

- Should all meetings take place at 1001 or should they be off-site?

- What space is available at 1001?

Incorporating this approach into this site

- For staff: Describe anything in the current program at 1001 that is similar to the Behavioral Activation approach.

Probe:

Do you think that BA would overlap with what clients receive in other interventions at 1001?

- For peers: Describe anything in your work already with patients that is similar to the Behavioral Activation approach.

- For staff: What types of roles do/have PRSs played in the methadone treatment program at 1001?

- What would be a reasonable number of clients for a PRS to work with if working full time and seeing clients weekly at 1001?

- Who do you think is the best supervisor for the peer working in this setting?

- For staff: *Who would be an appropriate point-of-contact for a PRS at 1001?*

-For staff: *During the research study, a study team member will supervise the PRS. If this work continues, after the study who would be an appropriate supervisor for a PRS at 1001?*

<u>Specific barriers and facilitators related to using PRSs for promoting MT retention</u>

- For PRSs: How do you include your personal story and "peer" identity into your work with clients?
    - What about when it differs from someone else's desired path to recovery? Please provide an example.

-What makes someone a good peer?

- What are challenges you anticipate with a peer role here that is focused on helping people engage in rewarding, meaningful activities?
    - What would make that easier?
- What are challenges you anticipate with a peer role here that is focused on helping people stay in treatment?
    - What would make that easier?

Closing questions/discussion

- What else would you like to share that may not have been covered in our conversation so far?

Patient Focus Group and Interview Guide

Note: This document is meant to be a guide for the facilitator to structure the focus groups and interviews; however, the facilitator is encouraged to follow the participants responses and probe to gather as accurate a sense of their perspectives as possible. The primary topic areas will stay the same, but the specific questions we expect to change based on information shared during the respective focus groups and interviews.

Feedback on a Behavioral Activation Intervention (Peer Activate)

We want to get your feedback on one idea we have for helping to keep people on treatment here at the treatment program. We think this is something that could be led by peers to support clients to get to successful treatment outcomes. The idea is that people are more likely to succeed when they're doing activities that are rewarding and enjoyable to them and do not involve alcohol or drugs. We're hoping to look at how well this works to have peers support clients to do these positive activities. We want to hear your thoughts on this idea.

Do you have any questions right now?

<u>**Adaptations to BA for setting**</u>

Activity ideas

- What things do you enjoy doing that don't involve substance use?
    Probe: *This can be things that you did a long time ago.*

- What are examples of other activities that people are able to do in the community that you think you may enjoy doing?

Barriers and facilitators to activity engagement
- What makes it difficult for you to do these activities?
- What makes it easier for you to do these activities?

Probe:
- *How can we make different substance-free activities easier and more accessible?*

Views on peer-led BA at MT initiation
- How might a peer support you to get involved in and do some of these substance free activities you're talking about? Can you give an example?
- How might it work to have peers do activities with you?

What are your thoughts on this type of program (that is – peers supporting clients in treatment by helping them do healthy, substance free activities) for clients starting methadone treatment at 1001?

Probes:
- *Who do you think this type of program would work best for?*
- *What level of motivation (readiness to recover) would clients need for this to work?*
- *Does this sound like something you would be interested in doing? Why or why not?*

Feedback on structure and format
- For this type of program, the peer usually meets with a client one-on-one and plans out activities with them. What do you think about one-on-one meetings versus group meetings?

Probes:
- *What about group meetings with peers (therapy)?*
- *What about group activities?*

- Usually this type of program includes about 10 or 12 meetings. What do you think about that length?
- How often would you want to meet with the peer? (i.e., once per week? More?) Why?

- When would be the best time for new clients to start working with a peer to schedule these positive activities? Why?

Probes:
-at MT initiation?
-after maintenance dose is reached?
-how long after T intake?

- How would scheduling peer meetings fit into your dosing schedule and other scheduled time in the methadone program (medical appointments, appointments with counselor, required group meetings)?

Probe:
- What does your typical day look like just after starting MT?

- Where should meetings happen?
  - What space makes you comfortable?
  - Should all meetings take place at 1001 or should they be off-site?

<u>Specific barriers and facilitators related to using PRSs for promoting MT retention</u>
- Have you ever worked with a peer in your recovery process?
  - If so, can you explain that experience for you?
  - What did you like and/or dislike about working with a peer?
- What qualities would you want in a peer?
- How would you want a peer to act around you?
- What would make you most comfortable working with a peer?
- Would you want other people to know you were working with a peer?
  - If no, why not? If yes, why?
- Would you be interested in working with a peer in your recovery process now?
  - If yes, why?
  - If no, who would you prefer to work with?

- Who at 1001 has been the most involved in supporting your recovery process?
- How would you want a peer to work with those other people who are involved in your treatment?

Closing questions/discussion
- What else would you like to share that may not have been covered in our conversation so far?

- What do you think would make the recovery experience better? Feel free to be creative!
