# Supplementary material for: “Sometimes you have to take the person and show them how”: adapting behavioral activation for peer recovery specialist-delivery to improve methadone treatment retention
Source: Subst Abuse Treat Prev Policy. 2023 Mar 6;18:15. doi: 10.1186/s13011-023-00524-3 (PMC9990281; doi:10.1186/s13011-023-00524-3)
Supplement: Supplementary file 2 — Additional file 2. Thematic framework. [file 13011_2023_524_MOESM2_ESM.pdf]

Appendix B

Thematic framework

Themes	Codes
Acceptability and appropriateness of PRS role	Shared lived experience
	Peer role boundaries / scope of peer work
	Desirable peer qualities
	PRS work with other members of the treatment team
Acceptability and appropriateness of BA	Boredom/ keeping busy
	Related content already being used in this setting
	Re-connecting to what makes you happy
Barriers to BA intervention in community-based MT	Balancing with other aspects of treatment
	Level of readiness or motivation
	Barriers to activity engagement
	Physical and mental health
Facilitators of PRS-delivered BA	Flexibility
	Timing of intervention
	Location
	Incentives
BA adaptations	Group versus individual intervention
	Peer leading/demonstrating activities
